# Partisan politics and stock returns under strong presidential regimes: Religion and politics

**DOI:** 10.1371/journal.pone.0359627

**Published:** 2026-10-30

**Authors:** Javier Mella

**Affiliations:** 1 Universidad de los Andes, Santiago, Chile; 2 Center for Advanced Transportation, Logistics, and Economic Competitiveness (CATLEC), Santiago, Chile; The University of Warwick, UNITED KINGDOM OF GREAT BRITAIN AND NORTHERN IRELAND

## Abstract

This study examines how partisan politics affects stock returns in Latin American countries characterized by strong presidential regimes. Using firm-level data, we show that the widely documented Democratic Party premium in the United States does not extend to this setting. Instead, stock returns are highest under centrist governments, while elections won by left-wing candidates trigger negative market reactions. Beyond the traditional left–right dimension, we introduce a novel religious classification of political parties. We find that markets perform significantly better when presidents are affiliated with Christian parties, with higher average returns and positive abnormal returns following electoral victories. These effects are accompanied by lower measures of risk and higher profitability, suggesting that the superior performance is not driven by risk premium but rather by improved expected cash flows and market underreaction to political outcomes. Overall, the results indicate that partisan effects on stock returns depend on institutional context and extend beyond the conventional ideological spectrum. The findings highlight the economic relevance of political religiosity and provide new evidence that financial markets respond systematically to political characteristics in strong presidential systems.

## 1. Introduction

This paper explores the effect of partisan politics on stock returns. This analysis examines a sample of countries with a high degree of disagreement on the main economic policies and a strong presidential system. The discrepancy in the economic policies is helpful to allow for enough variation among left-wing and right-wing parties or coalitions in office, and the strong presidential systems enable the premier to change the economic policies according to the party’s view. Moreover, this research also studies the potential effect of partisan politics on the religious spectrum.

The results show that firm-level stock returns are higher during Centrist party government terms compared to periods under left or right-wing government in a sample of Latin American countries. This is a monthly return of 1.90% for the periods under a Centrist president, compared to 1.59% (1.42%) for periods under right-wing (left-wing) governments. Moreover, for a government under a president from a Christian party, the firm-level stock returns have an average of 1.70%, compared to 1.50% for the periods during secular party governments. On average, the one-day market reaction after an election when a leftist candidate wins the ballot is negative and significantly lower than when a rightist candidate wins the election, a difference of 3.62% in daily returns. Similarly, the market reaction differential between a Christian and a Secular party candidate winning the election is 4.95%, with the market returns being higher when the former wins.

Further exploration of the previous results from a risk-based or cash flow-based explanation provides inconsistent results for the left-right wing dimension. For example, Economic Policy Uncertainty is lower during a left-wing government in Brazil but higher in Chile. For the panel data, Economic Policy Uncertainty is higher during right-wing governments. Regarding the cash flow-based explanation, profitability is used as a proxy and is lower under centrist presidencies.

For the Christian-Secular dimension, the performance is consistently better under Christian party governments. Lower Economic Policy Uncertainty for Chile, lower stock price crash risk, and higher profitability are observed when the president is from a Christian party. These results are consistent with cash flow-based explanation and underreaction.

This is the first paper that explicitly analyzes this religious dimension. Religious affiliation of political parties is expected to be economically relevant because it is associated with systematic differences in policy priorities, governance norms, and risk attitudes. Political parties with explicit religious roots, in particular Christian democratic traditions that have had some success in Latin America, have historically emphasized fiscal prudence, redistribution, social market principles, and institutional stability, in part due to the ability to build cross-class coalitions [1]; indeed [2,3], show that voting is shaped not only by class but by religion, even with differences among Christians. These previous policies can influence firms’ expected cash flows through taxation, regulation, and government spending priorities [4,5]. In addition, a growing literature links religiosity to economic behavior, showing that religious environments are associated with lower corporate risk-taking, more conservative financial policies, and improved governance [6–8]. These findings suggest that governments aligned with religious parties may shape both firm behavior and investor expectations by affecting perceived risk and governance quality. From an asset pricing perspective, differences in expected cash flows and discount rates linked to political ideology may therefore extend beyond the traditional left–right dimension. Consequently, examining the religious dimension provides a theoretically grounded channel through which political characteristics can affect financial markets. However, this differs from [9], which studied the effects of policy and policy uncertainty on innovation, specifically the left-right dimension, while only tangentially mentioning that the religious dimension could play a role.

In this study, a particular sample of Latin American countries is chosen due to the extensive literature that supports the notion that these nations are characterized by a strong presidential regime [10–12]. In the Latin American context, a “strong presidential system” typically refers to executives endowed with substantial formal constitutional authority, strength of presidents relative to the legislature, partisan leverage, and informal influence over the policy process. First, formal powers include decree authority, that is proactive power, total or partial veto power, that is reactive power, or exclusive bill initiation in some relevant areas, like government spending [11,13]. Second, the strength of presidents depends on executive versus legislative relations, particularly the extent to which institutional rules grant the executive control over the legislative agenda and constrain congressional power [14]. Third, partisan powers, this is likely the least consistent in Latin America but could enhance presidential influence in congress [15,16]. Last, a pattern described as “delegative democracy” [17] when there is personalistic leadership as it is found in some periods in Latin America.

While there is extensive evidence of the effect of partisan politics on stock returns in the U.S [18–23], the international evidence is less conclusive [24]. posit that the left-wing premium is exceptional and show that from a sample of 15 member countries in the Organisation for Economic Co-operation and Development (OECD), only Denmark, Germany, and the U.S. displayed this premium [25]. find that partisanship plays a role in the value relevance of corporate tax expenses.

For Latin America, the literature is limited [26]. found evidence of a positive market reaction in Chile when the right-wing presidential candidate increased his popularity in the political polls for the 2009 election [27]. show that political news affects volatility and trading volume in Argentina. However, they do not explore the asset returns in the Argentinian market.

Our empirical analysis uses firm-level data from Compustat Global for Brazil, Chile, Colombia, Mexico, and Peru is used. Moreover, the party’s classification mostly follows [28]. The empirical approach raises some concerns. First, it is never true in the sample that there are only two parties or groups of parties, one left-wing and one right-wing, that are relevant to the country’s political system [29]. postulate that the macroeconomic effects of party policy are more evident in a two-party system than in a multi-party system. Second, only a few shifts occur between parties—for instance, the same center-left coalition governed in Chile from 1990 to 2010. Third, a party can be challenging to classify on the left-right spectrum. For example, the electorally successful Peronism/Justicialism movement in Argentina has displayed both left and right-wing policies and has elected presidents from both subgroups [30]. Last, as [31] argued, the number of observations is limited. Thus, we pool all firm-level data from various countries, which differs from what [19] did.

This research sheds light on several issues. Firstly, this paper shows that the left-wing premium is exceptional and not observed in this sample. However, this paper confirms the importance of partisan politics on the stock markets in a different setting. For example, the significantly different market reaction after a left-wing candidate is elected compared to a right-wing elected presidential candidate. Besides, this research contributes to the effect of partisan politics on the stock markets, in this case, through the religious-secular dimension. That is, the returns are higher, and the risk is lower during periods with a president from a Christian party.

This paper also contributes to understanding the effect of religiosity on the stock price crash risk. Different from [7], which explores the religiosity of the firm’s members, this research shows that the religiosity of the party in office is correlated with the stock price crash risk at the firm level. Also, different from [32], which explores stock price crash risk in China, we use an international sample of Latin American countries.

Overall, this research differs from [19], as we use firm-level data aggregated at the cross-country level due to several reasons: the length of the data available is less than for the US, it also allows the exploration of crash risk that has been explored at the motivated at the religious dimension. However, it has limitations as we assume homogeneity in the economic situation, stock market structure, and other institutional variables.

The remainder of this study is divided into seven sections. Section 2 develops testable hypotheses. Section 3 describes the financial and political data used in the paper. Section 4 shows the main empirical results. Section 5 explores alternative explanations for the main empirical results. Section 6 includes the robustness analysis. Section 7 discusses the implications of empirical results. Finally, section 8 concludes the study.

## 2. Hypotheses development

In this section, we describe the potential explanations of how partisan politics affects stock returns for two different dimensions: the left-right wing dimension and the secular-Christian dimension. Once the hypotheses are outlined, they will be tested on a sample of Latin American countries.

### 2.1. A left-wing premium

The empirical literature has shown a Democratic Party premium for the U.S. According to [24], the international evidence is feeble for a left-wing premium.

On the theoretical side [22], argue that in periods of high risk-aversion, the Democrats (i.e., left-wing party candidates) are more likely to be elected, and the stock returns tend to be higher than when Republicans (i.e., right-wing party candidates) are elected. Their model also implies a positive reaction when a right-wing candidate wins the presidential election. One mechanisism showed in [33] is by increasing taxes in left-wing periods and redistributions, result that is also consistent with [23]. Another mechanisism to explain differences in returns is due to government spending, as shown in [21].

Based on the previous discussion, the following hypotheses are proposed:

H1: There is a left-wing premium in the sample of Latin American countries.
H2: There is a negative reaction when a leftist presidential candidate wins the election.

Note that if H1 and H2 were true, these results would be consistent with higher returns reflecting compensation for bearing higher risk.

### 2.2. A religious effect

The empirical results have supported the view that higher religiosity is correlated with lower exposure to risk as measured by volatility of stock returns [6] and more ethical behavior from managers as measured by stock price crash risk [7]. The underlying assumption is that governments under the presidency of a religious (i.e., Christian) party would lead to lower risk behavior from managers and, therefore, if investors perceive firms to be less risky under Christian-party governments, the required rate of return would decline, leading to an immediate positive stock price reaction following the election of a Christian-party candidate. Then, the immediate reaction after a Christian party candidate gets elected would be a one-time positive reaction, given the lower risk level. Another potential mechanism, based on [1], is the view that Christian Democracy parties can form cross-class coalitions and provide a better social climate, which allows for better economic performance and reinforces subsidiarity without the potential cost of increased government spending and deficit budgets, consistent with higher returns due to, for example, lower discount rates.

Thus, the following hypotheses are proposed:

H3: There is a lower risk premium when the president in office is from a Christian party.
H4: There is a positive reaction when a Christian party presidential candidate wins the election.

## 3. Data

### 3.1 Financial data

Firm-level financial data is from Compustat Global. Market-level financial data comes from Bloomberg. Macroeconomic variables come from the International Monetary Fund database.

To ensure data quality, a screening procedure is proposed. For a country to be included in the sample, it must have returns data available in the Global Compustat database, as this is our main analysis variable. Moreover, the country to be included must have at least five presidential elections during the sample period, and at least 50% of the period must be classified on the left-right spectrum, that is, neither classified as personalist nor as other bloc by [28], to ensure we have sufficient variation and presidential cycles. Finally, for the market reaction after an election analysis, the country needs to have the MSCI stock index for at least 10 years; thus, we can assure that several elections are included in the sample. Accordingly, the final selection includes Brazil, Chile, Colombia, Mexico, and Peru.

Also, the data does not include ADR’s, GDR’s, and stocks traded in denominations different from the local currency or US dollar. Due to the high inflation in part of the period, several countries changed their legal currency during the period for which data is available. Then, we exclude discontinued local currencies, for example, the Brazilian Cruzeiro, as this affect the reliability of the data.

The firm-level data covers the period from July 1994 to December 2017 for Brazil, January 1989 to December 2017 for Chile, August 1992 to December 2017 for Colombia and Peru, and April 1993 to December 2017 for Mexico. The sample is restricted by Compustat Global’s availability and the screening process that was previously described. The sample also includes MSCI stock indices, representatives of the same countries based on their availability. Exchange rates are from Bloomberg, and the risk-free return in US dollars is from Kenneth French’s website. Table 1 summarizes the data.

**Table 1:** Summary statistics.

Panel A.						
Country	Start date	End date	Firm-year observations	% of total firm-year observations	Returns in local currency	Returns in U.S. dollars
Brazil	07-1994	12-2017	55,196	53%	0.0125	0.0159
Chile	01-1989	12-2017	17,686	17%	0.0130	0.0153
Colombia	08-1992	12-2017	4,675	4%	0.0136	0.0159
Mexico	04-1993	12-2017	19,563	19%	0.0134	0.0182
Peru	08-1992	12-2017	7,377	7%	0.0202	0.0209
Total			104,497	100%		
Panel B.						
Country	First Election	Last Election	# of elections	% of total elections	Returns in local currency	Returns in U.S. dollars
Brazil	12-1989	10-2014	7	21%	-0.0073	-0.0128
Chile	12-1989	12-2017	7	21%	0.0226	0.0285
Colombia	06-1994	06-2018	7	21%	0.0170	0.0206
Mexico	07-1988	07-2018	6	18%	0.0164	0.0212
Peru	04-1995	06-2016	6	18%	-0.0056	-0.0033
Total				100%		

Panel A reports the summary statistics for firm-level data, and it covers the period from July 1994 to December 2017 for Brazil, January 1989 to December 2017 for Chile, August 1992 to December 2017 for Colombia and Peru, and April 1993 to December 2017 for Mexico. Panel B reports the summary statistics for country-level data, and it covers the elections from 1988 to 2018 for Brazil, Chile, and Mexico, and from 1994 to 2018 for Colombia and Peru. Returns in local currency and U.S. dollars are gross returns per month.

### 3.2 Political data

Classifying a president in the political space can be challenging in a two-party system (E.g., the U.S., the U.K., and Australia). However, the task is more challenging for a multi-party system standard in the sample of countries used in this research. To complicate this situation further, some parties can have volatile political platforms. Thus, the approach of this paper is to rely on previous research on Latin American politics. We updated the classification of Latin American political parties introduced by [28]. This is a comprehensive classification of the political parties using a different panel of experts for every country. The main drawback is that it has not been updated since its publication.

#### 3.2.1 Political ideology index: Presidential index.

[28] categorizes political parties from a group of Latin American countries based on the opinion of a panel of experts. Every party is classified into two dimensions: Christian or Secular and Left, Center, or Right. The combination of these two dimensions leads to six blocks. Parties not fitting into these six groups are classified as Other Bloc, Personalist, or Unknown. The updated classification based on [28] is included in the appendix, Table A1 in S1 File. The revised classification is based on the following criteria: i. Keep the party in the category from [28], ii. If the party is new, follow the party of origin of their founders/leaders, and finally, if needed, iii. Additional web sources.

## 4. Empirical results

### 4.1 Is there a left-wing premium?

[19] propose a regression including a stock index return and political dummies for the Democratic and Republican parties. Based on their work, we modified their regression using a firm-level regression for a set of countries. Moreover, a Center party classification is added to the Left and Right-wing parties. Then, the following regression is used to analyze the party in office’s effect on the stock returns.


$$\begin{array}{c} R_{i,t}=\gamma_{1}{LL}_{t}+\gamma_{2}{CC}_{t}{+\gamma }_{3}{RR}_{t}+\epsilon_{i.t}\;\forall 𝐭 \end{array}$$ (1)

$R_{i}$ refers to the firm i monthly returns for firm i, and LL, CC, and RR are the dummy variables for Left-wing, Center, and Right-wing parties. The results are described in Table 2. Also note that equations 1 and 2, including a set of control variables, related to the business cycle, are included in Appendix A2 and A3 in S1 File.

**Table 2:** Pooled-OLS regressions and the party of the President in office. Left to right dimension.

	Ri-Rf
VARIABLES	US dollars
LL	0.0142***
	(30.17)
RR	0.0159***
	(21.91)
CC	0.0190***
	(16.70)
LL-RR	−0.0017*
LL-CC	−0.0048***
RR-CC	−0.0031**
Control Variables	No

This table reports the results of the following pooled-OLS regressions. $R_{i,t}=\gamma_{\text{1}}{LL}_{t}+\gamma_{\text{2}}{CC}_{t}{+\gamma }_{\text{3}}{RR}_{t}+\epsilon_{i,t}$. $R_{i}$ refers to the firm i monthly excess returns in US dollars, and LL, CC, and RR are the dummy variables for Left-wing, Center, and Right-wing parties respectively. LL-RR, LL-CC, and RR-CC refer to differences in returns when the president is from a left-wing party and a right-wing party, and so on. The sample covers the period from July 1994 to December 2017 for Brazil, January 1989 to December 2017 for Chile, August 1992 to December 2017 for Colombia and Peru, and April 1993 to December 2017 for Mexico, according to data availability. The first row represents the coefficients; the second row reports the t-statistics. ***, **, and * indicate significance at the 1%, 5%, and 10% levels, respectively.

The results from Table 2 show that excess returns in US dollars are higher for the centrist parties when compared to left-wing and right-wing parties. The difference between centrist and left-wing governments is 0.48% per month, and between centrist and right-wing parties is 0.31% per month. Also, monthly returns are significantly higher for right-wing terms (1.59%) when compared to left-wing ones (1.42%). In unreported results, the differences are practically invariable for returns in local currency. Overall, these results do not support hypothesis one (H1).

Moreover, the Secular-Christian dimension is explored. To the best of our knowledge, this is the first paper that explicitly examines the effect of partisan politics in the religious dimension. The following regression is used to analyze this political aspect.


$$\begin{array}{c} R_{i,t}=\delta_{1}{SS}_{t}+\delta_{2}{XX}_{t}+\epsilon_{i.t}\;\forall 𝐭 \end{array}$$ (2)

$R_{i}$ refers to the firm i monthly returns for firm i, and SS and XX are the dummy variables for the Secular and Christian parties, respectively. The results are described in Table 3.

**Table 3:** Pooled-OLS regressions and the party of the President in office. Secular and Christian dimension.

	Ri-Rf
VARIABLES	US dollars
SS	0.0150***
	(37.64)
XX	0.0170***
	(15.47)
SS-XX	−0.0020*
Control Variables	No

This table reports the results of the following pooled-OLS regressions. $R_{i,t}=\delta_{\text{1}}{SS}_{t}+\delta_{\text{2}}{XX}_{t}+\epsilon_{i,t}$. $R_{i}$ refers to the firm i monthly excess returns in US dollars, and SS and XX are the dummy variables for Secular and Christian parties respectively. SS-XX refers to the differences in returns between secular and Christian party presidents. The sample covers the period from July 1994 to December 2017 for Brazil, January 1989 to December 2017 for Chile, August 1992 to December 2017 for Colombia and Peru, and April 1993 to December 2017 for Mexico, according to data availability. The first row represents the coefficients; the second row reports the t-statistics. ***, **, and * indicate significance at the 1%, 5%, and 10% levels, respectively.

Table 3 reports the novel results for excess returns for governments under Secular and Christian parties. The firm-level excess returns are, on average, 1.7% per month when a Christian party is in office, while the returns are 1.5% during periods with a Secular party president. The difference is a significant 0.2% per month. In unreported results, the differences are even more significant for returns in local currency; the difference is 0.41% and significant at the 1% level. Consequently, these results support for the rejection of hypothesis three (H3).

### 4.2 Is the market reaction after elections consistent with a risk-based explanation?

This section analyzes the stock market reaction after elections for the countries in our sample. According to the previous evidence for the U.S [19], there is expected to be a negative reaction after a left-wing candidate is elected.

There are 33 elections in the sample, of which the right-wing coalition won 17. When the two leading presidential candidates belonged to different versions of the same broad political spectrum (e.g., left versus center-left or right versus center-right), we classified them in relative terms. For example, in Brazil, the Workers’ Party (classified as left-wing by [28]) is coded as left-wing, whereas the Social Democratic Party (classified as center-left by [28]) is coded as right-wing because it represents the more right-leaning of the two main candidates in that election, even though it is center-left in absolute terms. This approach differs from the classification used in the previous section. There, parties are classified according to their absolute ideological position, whereas here they are classified according to their relative ideological position between the two leading presidential candidates. Nevertheless, the ideological classification remains based on [28].

The stock price reaction following the election is analyzed in relative terms between the two leading presidential candidates because, in several elections, both candidates belong to the same broad political spectrum but differ in their ideological positioning (e.g., left versus center-left). If a left-wing premium exists, the market reaction should become more pronounced as the winning candidate’s platform becomes relatively more left-wing than that of the main opponent. In other words, based on the previous results, a larger ideological difference between the two leading candidates should be associated with a stronger stock price reaction. Moreover, analyzing election outcomes in absolute ideological terms would require excluding a substantial number of elections, particularly in Brazil and Colombia, where the two leading candidates often belong to the same broad political spectrum.

The market reaction is measured the day after the election using MSCI stock indices in local currency and U.S. dollars.


$$\begin{array}{c} {After\_Election\_Return}_{\left(1\_day\right)}=\gamma_{1}LL+\gamma_{2}RR+\epsilon_{t} \end{array}$$ (3)

The results are presented in Table 4, including the OLS and Bootstrap approach to address the small sample size.

**Table 4:** Market reaction following the presidential election in Latin America.

	Ri	Ri	Ri-Rf	Ri-Rmk	Abnormal
VARIABLES	Local Currency	US dollars	US dollars	US dollars	Return (CAPM)
LL	−0.00602	−0.00770	−0.00780	−0.00550	−0.00626
	(−0.54)	(−0.54)	(−0.64)	(−0.49)	(−0.54)
RR	0.0228**	0.0285**	0.02837**	0.02995***	0.03000**
	(2.11)	(2.39)	(2.38)	(2.76)	(2.66)
LL-RR	−0.0288	−0.0362	−0.0362	−0.0355	−0.0363
*p-value OLS*	0.0729*	0.0423**	0.0425**	0.0300**	0.0324**
*p-value Bootstrapped*	0.0583*	0.0309**	0.0310**	0.0208**	0.0226**
					

The table reports the market reaction the day after the presidential election for the 33 presidential elections in the sample. The regression is:${After\_Election\_Return}_{\left(\text{1}\_day\right)}=\gamma_{\text{1}}LL+\gamma_{\text{2}}RR+\in_{t}$. LL and RR are the dummy variables for Left-wing and Right-wing parties respectively. *LL-RR* refers to differences in returns when the president is from a left-wing party and a right-wing party. ***, **, and * indicate significance at the 1%, 5%, and 10% levels, respectively.

The result in Table 4 is consistent with the market reaction observed in the U.S. when a Democratic presidential candidate wins the election. The difference in the market reaction the day after the election goes from 2.88% for returns in local currency to 3.62% for returns in U.S. dollars. All four different measures observe significant differences in the market reaction. In all cases, the response is negative when a left-wing candidate wins the election and positive when the right-wing candidate triumphs. Also, when the abnormal return is calculated using the CAPM model and the U.S. market return as a proxy for the market, the results are practically the same, with a difference of 3.63%. These results support hypothesis two (H2).

Similarly, the Secular-Christian dimension is explored for the day after the election. However, it is observed that in only four elections, the candidate of the Christian party ended up winning the ballot.


$$\begin{array}{c} {After\_Election\_Return}_{\left(1\_day\right)}=\delta_{1}SS+\delta_{2}XX+\epsilon_{t} \end{array}$$ (4)

The results in Table 5 are significant for the Secular-Christian dimension. The election of the Christian-party candidate is seen as good news, and the one-day market reaction is significantly higher when the outcome of the election favors a presidential candidate from a Christian party. The one-day return difference goes from 3.37% to 4.95%, always having higher returns when the non-secular candidate wins. The results are consistent with an expected improved performance during terms led by a Christian party member. Also, when the abnormal return is calculated using the CAPM model and the US market return as a proxy for the market, the results are practically invariant, with a difference of returns equal to 4.39%. Overall, these results are consistent with hypothesis four (H4).

**Table 5:** Market reaction following the presidential election in Latin America.

	Ri	Ri	Ri-Rf	Ri-Rmk	Abnormal
VARIABLES	Local Currency	US dollars	US dollars	US dollars	Return (CAPM)
SS	0.00429	0.00495	0.00484	0.00753	0.00710
	(0.51)	(0.54)	(0.52)	(0.88)	(0.80)
XX	0.0417*	0.0545**	0.05427**	0.05070**	0.05098**
	(1.84)	(2.19)	(2.18)	(2.20)	(2.13)
SS-XX	−0.0374	−0.0495	−0.0494	−0.0432	−0.0439
*p-value OLS*	0.131	0.0718*	0.0721*	0.0891*	0.0958*
*p-value Bootstrapped*	0.0008***	0.0003***	0.0004***	0.0005***	0.0005***

The table reports the market reaction the day after the presidential election for the 33 presidential elections in the sample. The regression is${After\_Election\_Return}_{\left(\text{1}\_day\right)}=\delta_{\text{1}}SS+\delta_{\text{2}}XX+\in_{t}$. SS and XX are the dummy variables for Secular and Christian parties respectively. SS-XX refers to the differences in returns between secular and Christian party presidents. ***, **, and * indicate significance at the 1%, 5%, and 10% levels, respectively.

We argue that the election result is the key information in the market at that time and it should be absorbed immediately, and reflected in one day, as the efficient market hypothesis suggests. Moreover, the event is precisely dated and by adding more post-election days we risk contamination from other news. However, as suggested by a reviewer, we repeated a similar analysis, using cumulative returns in local currency and in US dollars, for 2, 3, 4 days and a week after the election, and we found that the signs of the different specifications remain, but the statistical significance disappears in several cases for the left-right dimension but remain significant for the Christian-secular dimension. Results are available upon request.

## 5. Risk-based and Cash-flow-based explanations

The results in this section show potential explanations for the differences in returns for periods with different parties in office. The risk-based explanation and the cash-flow-based explanation are analyzed. The possible sources of risk discussed in this paper are the Economic Policy Uncertainty Risk, the Stock Price Crash Risk, and the volatility risk. On the other hand, the cash-flow-based explanation is discussed by contrasting the return on equity (ROE) during the different presidential terms.

### 5.1 Economic policy uncertainty risk

One of the potential explanations for the Democratic Party (left-wing) premium in the U.S. is a risk premium given a higher level of economic policy uncertainty (EPU). This possible explanation is explored for the left-right and Christian-secular dimensions in a sample of Latin American countries. To analyze this idea, Economic Policy Uncertainty indices based on [34] for Mexico, Brazil, Chile, and Colombia are used. Note that, for the Chilean and Colombian indices, details can be found in [35] and [36], respectively. All these indices are obtained from the policy uncertainty project’s website (www.policyuncertainty.com). According to the previous empirical results, for the case of an Economic Policy Uncertainty Risk, a higher level should be expected for center-wing and Christian party presidencies; thus, that premise is explored.

The results in Table 6, panel A, show that for Brazil, the EPU is significantly higher during Centrist party presidencies compared to left-wing presidencies. For Chile, left-wing governments show higher EPU compared to center and right-wing presidencies. For Colombia, the EPU is higher during right-wing presidencies compared to Centrist party governments. When all the data is combined in a panel, the EPU is significantly higher under right-wing parties compared to center and left-wing parties. The results are mostly inconsistent across countries, so it is impossible to find support for a risk premium.

**Table 6:** Economic Policy Uncertainty index (EPU) across Latin America.

	Brazil	Chile	Colombia	Mexico	Panel
VARIABLES	1991-2017 with gaps	1993-2017	1994-2016	1996-2017	1991-2017 country F.E with gaps-unbalanced
**Panel A: Left-Right dimension**					
LL	119.90***	112.26***	–	–	131.80***
	(24.97)	(30.87)	–	–	(30.97)
RR	–	87.29***	122.53***	98.39***	162.19***
	–	(12.91)	(23.60)	(22.70)	(16.88)
CC	334.10***	87.02***	75.42***	–	140.69***
	(17.26)	(17.22)	(13.91)	–	(16.71)
**LL-RR**	**–**	24.97***	**–**	**–**	−30.39***
**LL-CC**	−214.20***	25.24***	**–**	**–**	**−8.89**
**RR-CC**	**–**	**0.27**	47.11***	**–**	21.50***
					
**Panel B: Secular-Christian dimension**					
SS	132.32***	106.66***	100.00***	98.63***	100.00***
	(23.86)	(32.77)	(24.98)	(15.31)	(23.52)
XX	**–**	87.02***	**–**	98.20***	90.27***
	**–**	(16.95)	**–**	(16.70)	(11.91)
**SS-XX**	**–**	19.64***	**–**	**0.43**	**9.73**
					
# of Observations	276	300	276	264	1,116

Table 6 reports the Economic Policy Uncertainty (EPU) index across Latin America, including: Brazil, Chile, Colombia, and Mexico. LL, CC, and RR are the dummy variables for Left-wing, Center, and Right-wing parties respectively. SS and XX are the dummy variables for Secular and Christian parties correspondingly. *LL-RR, LL-CC,* and *RR-CC* refer to differences in returns when the president is from a left-wing party and a right-wing party, and so on. *SS-XX* refers to the differences in returns between secular and Christian party presidents. ***, **, and * indicate significance at the 1%, 5%, and 10% levels, respectively.

The results in Table 6, panel B, show that for Chile, the Christian party governments have lower EPU compared to secular governments. For Mexico, there are no significant differences between Secular and Christian presidencies. When all the data is combined in a panel, the EPU is significantly higher when a secular party is in office compared to when a Christian party is in office. Altogether, the results do not show a Christian risk premium; therefore, they are inconsistent with the risk-based explanation for the Christian party presidencies.

### 5.2 Stock price crash risk

Another potential explanation for the differences in the returns among different parties in office is differences in the level of crash risk. There is evidence that shows a negative correlation between religiosity and stock price crash risk at the firm level [7]. While [7] hypothesize that religion makes withholding bad news by the firm’s executives less likely, we speculate that a government under a Christian party will act similarly. In turn, this makes it more likely for firms to follow the social norms encouraged by the government and consequently lower the stock price risk at the firm level.

In order to calculate the two measures of firm-specific stock price crash risk, the following regression is estimated:


$$\begin{array}{c} r_{j,t}=\alpha_{j}+\beta_{j}r_{m,t-1}{+\gamma }_{j}r_{m,t}{+\delta }_{j}r_{m,t+1}+\epsilon_{j,t} \end{array}$$ (5)

$r_{j,t}$ refers to the return on stock j on day t, and $r_{m,t}$ refers to the return on the stock market in the specific country according to the MSCI index in US dollars. The regression is calculated separately for the Chilean and Mexican markets. 1-day lead and lag terms are added in case of any non-synchronous issue. From equation 5, the residual is obtained, and then the firm-specific daily return is calculated as $R_{j,t}=(1+residual)$, which is the natural logarithm of one plus the residual.

Two measures of crash risk used commonly at the firm level (see, for example [7]) are modified for this research. The measures are the negative coefficient of skewness of firm-specific daily returns (NCSKEW) and the down-to-up volatility (DUVOL). The NCSKEW is calculated as follows:


$$\begin{array}{c} {NCSKEW}_{j,T}=\frac{-\left(n{\left(n-1\right)}^{3/2}\sum R_{j,t}^{3}\right)}{\left(\left(n-1)(n-2\right){(\sum R_{j,t}^{2})}^{3/2}\right)} \end{array}$$ (6)

${NCSKEW}_{j,T}$ refers to the negative coefficient of skewness of firm-specific daily returns for firm j and year T. n refers to the number of observations per firm and year. The DUVOL measure is calculated as follows:


$$\begin{array}{c} {DUVOL}_{j,T}=log\left[\frac{(n_{u}-1)\sum_{down}R_{j,t}^{2}}{(n_{d}-1)\sum_{up}R_{j,t}^{2}}\right] \end{array}$$ (7)

${DUVOL}_{j,T}$ refers to the down-to-up volatility for firm j and year T. $n_{u}$ refers to the number of observations with returns higher than the mean per firm and year. $n_{d}$ refers to the number of observations with returns lower than the mean per firm and year. Equation 8 shows the relationship between the measures of crash risk and the party of the president in office in the Secular (SS) and Christian (XX) dimensions. As the regression is annual, the convention regarding the political variable is to use the president who was in office at the beginning of the year, and this is after 15 trading days.


$$\begin{array}{c} {Crash\_Risk}_{j,T}=\delta_{1}{SS}_{T}+\delta_{2}{XX}_{T}+\epsilon_{j} \end{array}$$ (8)

Table 7 reports the results from Equation (8), using NCSKEW and DUVOL to measure stock price crash risk for Chile and Mexico. The impact of the ruling party on crash risk differs significantly between Christian and Secular periods. The differences in the coefficients for periods in which the president belongs to a Christian party (XX) relative to periods under a Secular party president (SS) are generally negative and statistically significant at the 1% level. Likewise, all crash risk measures, except DUVOL in Mexico, are lower during periods governed by a Christian party. The DUVOL measure for Mexico shows no statistically significant difference across political regimes.

**Table 7:** Stock price crash risk: The case of Chile and Mexico.

	Chile		Mexico	
	NCSKEW	DUVOL	NCSKEW	DUVOL
XX	−1.47***	−0.23***	−0.74***	−0.10***
SS	−1.10***	−0.09***	−0.68***	−0.14***
XX-SS	−0.37***	−0.14***	−0.06	0.04

Table 7 reports the Stock Price Crash Risk across in Chile and Mexico. The NCSKEW refers to the negative coefficient of skewness of firm-specific daily returns and is calculated as ${NCSKEW}_{j,T}=\frac{-\left(n(n-\text{1}{)}^{\text{3}/\text{2}}\sum R_{j,t}^{\text{3}}\right)}{\left((n-\text{1})(n-\text{2}){\left(\sum R_{j,t}^{\text{2}}\right)}^{\text{3}/\text{2}}\right)}$ and the DUVOL refers to the down-to-up volatility (DUVOL) and is calculated as ${DUVOL}_{j,T}=log\left[\frac{(n_{u}-\text{1})\sum_{down}R_{j,t}^{\text{2}}}{(n_{d}-\text{1})\sum_{up}R_{j,t}^{\text{2}}}\right].$ SS and XX are the dummy variables for Secular and Christian parties respectively. *SS-XX* refers to the differences in returns between secular and Christian party presidents. ***, **, and * indicate significance at the 1%, 5%, and 10% levels, respectively.

One possible explanation for the higher stock returns observed during Christian-party presidencies is that investors require compensation for bearing greater risk. If this were the case, higher returns should be associated with higher stock price crash risk. However, our results indicate the opposite. Crash risk is generally lower during Christian-party presidencies, suggesting that the superior stock returns documented in earlier sections cannot be attributed to a risk premium associated with higher stock price crash risk. Instead, the evidence indicates that investors earn higher returns despite facing lower crash risk under Christian-party governments.

### 5.3 Volatility

This section includes a test to examine the hypothesis of higher returns due to compensation for risk. The higher returns when Christian parties are in office might be explained by these different policies between Christian and Secular parties. Similarly, the same hypothesis is tested to explore a left-wing premium. GARCH and EGARCH models are used with daily returns and partisan variables. Please note that GARCH and EGARCH models are retained with the constant terms to avoid any issues with the correct specification of the conditional variance equations. Also, the daily volatility computed from within-month daily returns is used.

#### 5.3.1 GARCH and EGARCH models.

The results in Table 8 panel A from the GARCH model show that periods under left-wing presidents are related to higher returns for Chile and Peru when compared to right-wing presidential periods. For Colombia, the returns are higher for periods under right-wing presidents than for periods with a centrist president. Similarly, volatility tends to be lower for left-wing presidential periods in Brazil, Chile, Colombia, and Peru. Therefore, there is no left-wing risk premium due to volatility. The results in Table 8 panel B from the EGARCH model show that periods under a leftist president are related to higher returns for Brazil when compared to right-wing presidential periods. In all cases, under left-wing or centrist presidents, the markets tend to be less volatile. Therefore, no left-wing risk premium explained by volatility is observed.

**Table 8:** Stock Price Volatility risk and the left-right wing political dimension across Latin America.

Panel A				
	Country 2 Brazil – GARCH (1,1)	Country 3 Chile – GARCH (1,1)	Country 4 Colombia – GARCH (1,1)	Country 6 Peru – GARCH (1,1)
***Constant – (β_0_)***	0.0015***	0.0003**	0.0002	0.0011***
***LL***	-0.0005	0.0004*	NA	-0.0005
***CC***	-	-	-	-
***RR***	NA	-0.0001	0.0010***	-0.0008*
**ARCH - α_1_**	0.0923***	0.1346***	0.2126***	0.0761***
**GARCH - γ_1_**	0.8943***	0.8250***	0.7368***	0.9052***
***Constant***	-11.0703***	-11.8492***	-11.435***	-12.4781***
***LL***	-0.7635***	-0.0018	NA	0.4619***
***CC***	-	-	-	-
***RR***	NA	0.0921	0.4831***	0.5335***
**Panel B**				
	**Country 2** **Brazil – EGARCH (1,1)**	**Country 3** **Chile – EGARCH (1,1)**	**Country 4** **Colombia – EGARCH (1,1)**	**Country 6** **Peru – EGARCH (1,1)**
***Constant – (β_0_)***	0.0012**	0.0004***	0.0002	0.0009***
***LL***	-0.0007	0.0003	NA	-0.0004
***CC***	-	-	-	-
***RR***	NA	0.0001	0.0008***	-0.0004
**EARCH - α_1_**	-0.0608***	-0.0232***	-0.0532***	-0.0235***
**EARCH-A α_2_**	0.1766***	0.2503***	0.3778***	0.1578***
**GARCH - γ_1_**	0.9748***	0.9537 ***	0.9136***	0.9795***
***Constant***	-0.1691***	- 0.3972***	-0.7453***	-0.1667***
***LL***	-0.0237***	-0.0074**	NA	0.0062**
***CC***	-	-	-	-
***RR***	NA	0.0063*	0.0411***	0.0095***

Table 8 Panel A reports the Stock Price Volatility across Latin America, including Brazil, Chile, Colombia, and Peru. The results are obtained using a GARCH (1,1) model with the party in office classification in the Right-Left wing dimension as an independent variable. In all the cases, the Centrist Party variable is dropped because of collinearity. Panel B reports identical variables using an EGARCH (1,1) model. NA refers to not applicable; that is, there is no president of that party for the sample period.

Table 9 panel A results from the GARCH model show no differences in the mean and volatility equations between Christian and Secular presidencies. For the EGARCH model in panel B, there are no differences observed in Chile; however, there is lower volatility in the stock market for periods under a president from a Christian party in Mexico. Thus, there is no evidence of a risk-premium obtained during Christian party presidencies in Latin America.

**Table 9:** Stock Price Volatility risk and the secular political dimension across Latin America.

Panel A		
	Country 3 Chile – GARCH (1,1)	Country 5 Mexico – GARCH (1,1)
Constant **-(**$\beta_{0}$)	0.0006***	0.0010***
XX	−0.0003	0.0002
**ARCH –** $\alpha_{1}$	0.1351***	0.1244***
**GARCH –** $\gamma_{1}$	0.8242***	0.8431***
Constant	−11.8135***	−11.4759***
XX	−0.0288	−0.0965
**Panel B**		
	**Country 3** **Chile – EGARCH (1,1)**	**Country 5** **Mexico – EGARCH (1,1)**
Constant **-(**$\beta_{0}$)	0.0007***	0.0007***
XX	−0.0002	0.00002
**EARCH –** $\alpha_{1}$	−0.0225***	−0.0791***
**EARCH-A** $\alpha_{2}$	0.2544***	0.2000***
**GARCH –** $\gamma_{1}$	0.9528***	0.9681***
Constant	−0.4081***	−0.2546***
XX	0.0030	−0.0072***

Table 9 Panel A reports the Stock Price Volatility across Latin America, including Chile and Mexico. The results are obtained using a GARCH (1,1) model with the party in office classification in the Secular-Christian dimension as an independent variable, respectively. In all the cases, the Secular variable is dropped because of collinearity. Panel B reports identical variables using an EGARCH (1,1) model.

#### 5.3.2 Volatility – Standard deviation of within-month daily returns.

The volatility computed from within-month daily returns of the MSCI stock index in US dollars for each country in the sample is used. To test if the higher returns under a specific party are due to risk compensation, we examine the volatility of returns under different presidencies. Table 10 panel A shows the results from the regression:

**Table 10:** Volatility risk and the political dimensions across Latin America.

Panel A	
VARIABLES	Volatility
LL	0.0186***
	(486.42)
RR	0.0146***
	(248.50)
CC	0.0140***
	(151.92)
LL-RR	0.0040***
LL-CC	0.0046***
RR-CC	0.0006***
**Panel B**	
VARIABLES	Volatility
SS	0.0174***
	(533.91)
XX	0.0141***
	(155.91)
SS-XX	0.0033***

Table 10 reports the volatility across Latin America, including Brazil, Chile, Colombia, and Mexico. Panel A shows the results for the left-right wing dimension. LL, CC, and RR are the dummy variables for Left-wing, Center, and Right-wing parties respectively. Panel B shows the results for the religious dimension. SS and XX are the dummy variables for Secular and Christian parties correspondingly. *LL-RR, LL-CC*, and *RR-CC* refer to differences in returns when the president is from a left-wing party and a right-wing party, and so on. *SS-XX* refers to the differences in returns between secular and Christian party presidents. ***, **, and * indicate significance at the 1%, 5%, and 10% levels, respectively.


$$\begin{array}{c} {VOL}_{t}=\gamma_{1}{LL}_{t}+\gamma_{2}{CC}_{t}{+\gamma }_{3}{RR}_{t}+\epsilon_{t} \end{array}$$ (9)

The volatility is higher when the president is from a left-wing party. The volatility of daily returns is 1.86% during left-wing governments, compared to 1.46% and 1.40% during right-wing and center parties, respectively.

Table 10 panel B shows the results from the regression:


$$\begin{array}{c} {VOL}_{t}=\delta_{1}{SS}_{t}+\delta_{2}{XX}_{t}+\epsilon_{t} \end{array}$$ (10)

The volatility is higher when the president is from a secular party. The volatility of daily returns is 1.74% during periods when the government is from a secular party, compared to 1.41% during periods when the president is from a Christian party. These results are coincident with other sources of risk: the Economic Policy Uncertainty index and the Crash Risk, which are higher during periods when the president is from a secular party, compared to periods when the president ruling the country is from a Christian party.

### 5.4 Profitability

The disparity in returns may be due to differences in cash flows during presidential terms with presidents from different parties. For this explanation to be plausible, underreaction is needed to the good news of a president being elected from a party that would encourage an increase in the profitability of the firms in the economy. To investigate this potential explanation, we can see the results from the following panel regression with firm fixed effects:


$$\begin{array}{c} {ROE}_{i,t}=\sum \rho_{k}P_{k}+\pi^{\prime }X_{i,t}{++\varphi {ROE}_{i,t-12}+\epsilon }_{i,t}\;\forall 𝐭 \end{array}$$ (11)

${ROE}_{i}$ refers to the firm i return on equity measured as net income divided by book equity value, $P_{k}$ is the dummy variable for the political party in office k, and $X_{i}$ is the vector of control variables. The selection of these control variables follows [37] and includes the market-value-of-assets to book-value-of-assets ratio (MB), the dividend-payments to book-equity value ratio (DB), and a dummy for dividend-paying firms (DIV). The results in Table 11, Panel A, show that ROE is significantly lower in periods when the president is from a Centrist party compared to periods under a left-wing president. In contrast, there is no difference in profitability between left- and right-wing presidential terms. Then, it is not possible to explain the higher returns during centrist parties’ terms due to higher cash flows within those terms, using the ROE accounting measure as a proxy for the cash flows.

**Table 11:** Profitability across different political parties in office.

Panel A	
VARIABLES	Coefficient
ROE	0.0077***
	(7.99)
DB	0.8250***
	(58.14)
DIV	−0.0871***
	(−3.26)
MB	−2.62
	(−1.14)
LL	0.1345***
	(3.61)
RR	0.1347***
	(3.62)
CC	0.1275***
	(3.41)
**LL-RR**	**−0.0002**
**CC-LL**	−0.0070**
**CC-RR**	**−0.0072**
**Panel B**	
VARIABLES	Coefficient
ROE	0.0077***
	(8.02)
DB	0.8258***
	(58.26)
DIV	−0.0881***
	(−3.30)
MB	−2.54
	(−1.10)
XX	0.1719***
	(4.55)
SS	0.1356***
	(3.69)
**XX-SS**	0.0363***

This table reports the results of the following panel regressions with firm fixed effects. ${ROE}_{i,t}=\sum \rho_{k}P_{k}+\varphi {ROE}_{i,t-\text{1}\text{2}}{+\epsilon }_{i,t}$. The sample covers the period from July 1994 to December 2017 for Brazil, January 1989 to December 2017 for Chile, August 1992 to December 2017 for Colombia and Peru, and April 1993 to December 2017 for Mexico. The dependent variable is the ROE, the explanatory variables include the party of the president in office, twelve-month lag of the ROE, market-value-of-assets to book-value-of-assets ratio (MB), the dividend-payments to book-equity value ratio (DB) and a dummy for dividend-paying firms (DIV). Panel A shows the results for the left (LL), center (CC) and right (RR) political party. LL-RR, LL-CC, and RR-CC refer to differences in returns when the president is from a left-wing party and a right-wing party, and so on. Panel B shows the results for the Christian (XX) and Secular (SS) political dimension. SS-XX refers to the differences in returns between secular and Christian party presidents. ***, **, and * indicate significance at the 1%, 5%, and 10% levels, respectively.

The results in Table 11, Panel B, show that ROE is significantly higher during periods under a president from a Christian party compared to periods under a president from a Secular party. In contrast, the higher returns during periods under a president from a Christian party seem to be explained by underreaction to higher levels of cash-flows.

## 6. Robustness analysis of the main results and additional institutional aspects

### 6.1 Robustness analysis

In this section, we perform a robustness analysis of the main results, that is, Tables 2 and 3. First, we split the database into before and after the subprime crisis. The main results, the ones in Tables 2 and 3, are mostly consistent in magnitude and sign. However, the statistical significance is lost in two of the eight combinations (Tables 12–14).

**Table 12:** Panel A and B: Pooled-OLS regressions and the party of the President in office. Left-to-right dimension.

Panel A: Before the end of the subprime crisis (1987–2009)	
	Ri-Rf
VARIABLES	US dollars
LL	0.0200***
	(27.96)
RR	0.0242***
	(21.15)
CC	0.0257***
	(12.81)
LL-RR	−0.0042***
LL-CC	−0.0057***
RR-CC	−0.0015
Control Variables	No
Panel B: After the end of the subprime crisis (2010–2017)	
	Ri-Rf
VARIABLES	US dollars
LL	0.0105***
	(17.36)
RR	0.0109***
	(12.15)
CC	0.0173***
	(13.51)
LL-RR	−0.0004
LL-CC	−0.0064***
RR-CC	−0.0068***
	
Control Variables	No

This table reports the results of the following pooled-OLS regressions. $R_{i,t}=\gamma_{1}{LL}_{t}+\gamma_{2}{CC}_{t}{+\gamma }_{3}{RR}_{t}+\epsilon_{i,t}$. $R_{i}$ refers to the firm i monthly excess returns in US dollars, and LL, CC, and RR are the dummy variables for Left-wing, Center, and Right-wing parties respectively. *LL-RR, LL-CC,* and *RR-CC* refer to differences in returns when the president is from a left-wing party and a right-wing party, and so on. The sample covers the period from July 1994 to December 2017 for Brazil, January 1989 to December 2017 for Chile, August 1992 to December 2017 for Colombia and Peru, and April 1993 to December 2017 for Mexico, according to data availability. The first row represents the coefficients; the second row reports the t-statistics. ***, **, and * indicate significance at the 1%, 5%, and 10% levels, respectively. Panel A: t-statistics in parentheses. *** p < 0.01, ** p < 0.05, * p < 0.1. Panel B: t-statistics in parentheses. *** p < 0.01, ** p < 0.05, * p < 0.1

**Table 13:** Panel C and D: Pooled-OLS regressions and the party of the President in office. Secular and Christian dimension.

Panel C: Before the end of the subprime crisis (1987–2009)	
	Ri-Rf
**VARIABLES**	**US dollars**
SS	0.0215***
	(33.72)
XX	0.0218***
	(15.55)
SS-XX	−0.0003
	
Control Variables	No
Panel D: After the end of the subprime crisis (2010–2017)	
	Ri-Rf
VARIABLES	US dollars
SS	0.00537***
	(11.16)
XX	0.0113***
	(5.818)
SS-XX	−0.0059***
	
Control Variables	No

This table reports the results of the following pooled-OLS regressions. $R_{i,t}=\delta_{\text{1}}{SS}_{t}+\delta_{\text{2}}{XX}_{t}+\epsilon_{i,t}$. $R_{i}$ refers to the firm i monthly excess returns in US dollars, and SS and XX are the dummy variables for Secular and Christian parties respectively. *SS-XX* refers to the differences in returns between secular and Christian party presidents. The sample covers the period from July 1994 to December 2017 for Brazil, January 1989 to December 2017 for Chile, August 1992 to December 2017 for Colombia and Peru, and April 1993 to December 2017 for Mexico, according to data availability. The first row represents the coefficients; the second row reports the t-statistics. ***, **, and * indicate significance at the 1%, 5%, and 10% levels, respectively. Panel C: t-statistics in parentheses. *** p < 0.01, ** p < 0.05, * p < 0.1. Panel D: t-statistics in parentheses. *** p < 0.01, ** p < 0.05, * p < 0.1.

**Table 14:** Pooled-OLS regressions and the party of the President in office. Secular and Christian dimension.

	Ri-Rf
VARIABLES	US dollars
Constant: a	0.0223***
	(37.64)
XX	−0.0021***
	(−3.61)
Control Variables	No

This table reports the results of the following pooled-OLS regressions. $R_{i,t}=a+\delta_{\text{3}}{XX}_{t}+\epsilon_{i,t}$. $R_{i}$ refers to the firm i monthly excess returns in US dollars, and XX is a continuous variable regarding religiosity, specifically, the higher the value of XX, the lower the religiosity of the party. The sample covers the period from July 1994 to December 2017 for Brazil, January 1989 to December 2017 for Chile, August 1992 to December 2017 for Colombia and Peru, and April 1993 to December 2017 for Mexico, according to data availability. The first row represents the coefficients; the second row reports the t-statistics. ***, **, and * indicate significance at the 1%, 5%, and 10% levels, respectively.

Second, as suggested by a referee, we use the database of V-Dem initiative (https://v-dem.net/data/v-party-dataset/) [38]. We follow item “v2parelig”, which refers to the question “To what extent does this party invoke God, religion, or sacred/religious texts to justify its positions?”. Values 0, 1, 2, 3, and 4, are classified as always or almost always, often, about half of the time, rarely, and never, respectively. Consequently, we replaced the religiosity dummy variable with a continuous variable while keeping all the other variables and sample size unchanged from the previous analysis, finding significant results that are consistent with the previous one, that is, the higher the variable (i.e., more secular), the lower the return. As a robustness test, we use the V-Party “economic left-right scale” measure (item v2pariglef), which yields qualitatively consistent results, with centrist parties exhibiting superior stock market performance. We thank an anonymous reviewer for this suggestion. The results are reported in Table 15. More specifically, we construct two variables, *score1* and *score2*. The variable *score1* measures the ideological distance from the left to the center, where parties classified from 0 to 2 are considered left-wing and 3 represents the center. The variable *score2* measures the ideological distance from the center (classified as 3) to the right, where parties classified from 4 to 6 are considered right-wing. Consistent with our previous findings, we expect a positive coefficient on score1 and a negative coefficient on *score2*. In other words, stock returns should increase as political parties move toward the ideological center from either direction. The results reported in Table 15 are consistent with those presented in Table 2 and provide additional support for Hypothesis 3 (H3).

**Table 15:** Pooled-OLS regressions and the party of the President in office. Left to Right dimension.

	Ri-Rf
VARIABLES	US dollars
Constant: a	−0.0089***
	(−3.38)
Score1 (L to C)	0.0101***
	(8.63)
Score2 (C to R)	−0.0019**
	(−2.54)
Control Variables	No

This table reports the results of the following pooled-OLS regressions. $R_{i,t}=a+\delta_{\text{1}}{Score}_{\text{1}}+\delta_{\text{2}}{Score}_{\text{2}}+\epsilon_{i,t}$. $R_{i}$ refers to the firm i monthly excess returns in US dollars, and ${Score}_{\text{1}}$ and ${Score}_{\text{2}}$ are continuous variable regarding politics based on the political continuous variable from the V-party project called v2pariglef that goes from 0 (far left), to 3 (centrist), to 6 (far right). Thus, ${Score}_{\text{1}}$ the distance from left (classified from 0 to 2), to center and ${Score}_{\text{2}}$, the distance from the center to the right. The sample covers the period from July 1994 to December 2017 for Brazil, January 1989 to December 2017 for Chile, August 1992 to December 2017 for Colombia and Peru, and April 1993 to December 2017 for Mexico, according to data availability. The first row represents the coefficients; the second row reports the t-statistics. ***, **, and * indicate significance at the 1%, 5%, and 10% levels, respectively.

Third, for the placebo test, we randomly use two (XX, SS) or three (LL, CC, RR) dummies for the main regressions. As expected, we did not find any significant difference when proceeding with the placebo test.

Fourth, for Mexico, we exclude the period before the 2000 transition for the main regressions in Tables 2 and 3. The results are very similar to the regression with the whole sample. All significant results at 1% and 5% remain in direction, and the magnitude show a very small change. We thank an anonymous reviewer for this suggestion; results are available from the corresponding author upon request.

### 6.2 Additional institutional aspects

First, the congress and its control have had mixed results in previous research. For the US, while [19] found that the party controlling Congress is not relevant [23], found that the effect of corporate taxes on stock returns is mainly related to the control of the White House and Congress. The issue is not present, of course, in parliamentary systems. Thus, we explore some relevant matters, for example, the control of Congress by the party/coalition of the president. This is relevant, as many key economic measures, such as the budget and taxes, require congressional support. The main results from Tables 2 and 3 are robust to the inclusion of this control. The results for the control itself are not significant. These results are available from the corresponding author upon request.

Second, the results for the control of Congress interacting with the party are indeed significant for the left-to-right spectrum; however, they are not significant for the religiosity classification. It seems that the control of Congress is relevant, especially for right-wing presidents. The control of Congress adds to the right-wing president’s period a return of about 1.2% per month, while for left-wing and centrist presidencies, controlling Congress and the presidency reduces the return by about 0.38% and 0.48%, respectively. For the control of Congress by right-wing and left-wing parties, the results are significant at the 1% level, while for centrist control, the significance level is 10%.

Third, another issue we explore, related to Congress, is the presence of diverse societal views. As mentioned earlier, the case in Latin America is far from being a two-party system. However, it is still relevant to differentiate what would be the case with a more diluted power of the parties in Congress, which could lead to difficulties in finding middle-ground agreements necessary to make changes in key economic elements. Generally, there has been an increase in the number of parties or coalitions among the countries in the sample. The results showed a very small effect on the number of parties, which is economically insignificant, of about 0.2–0.3% per year.

Fourth, one of the key elements that has emerged over the last few decades is the independence of central banks. In particular, we investigate whether this independence influences stock returns and whether this institutional factor impacts the relevance of partisanship. We found no evidence that central bank independence explains stock returns. This result is most likely related to the fact that there is little variation, as most countries in the sample have granted independence to the monetary authority just before the beginning of the sample (Colombia), just after the beginning of the sample (Chile, Peru, and Mexico) or after the end of the sample (Brazil).

## 7. Discussion of the empirical results

Finally, a discussion regarding the drawbacks of the examined analysis and proposed further research is needed. First, the short period of the study, the high inflation during part of the sample, and the low variability in the political variable make further research needed. As time passes and more data becomes available, the tests will become more powerful.

The results are consistent with [24] in that the left-wing premium is exceptional, as they show that from a sample of 15 member countries in the Organisation for Economic Co-operation and Development (OECD), only Denmark, Germany, and the U.S displayed this premium. The political system differs greatly from most OECD countries, so this is a pertinent outcome.

An interesting outcome is that stock returns have lower volatility and higher returns during governments under a centrist party compared to periods when the president is from a right-wing party. Thus, if the Democratic Party of the U.S. is considered a centrist party, as in [39], the results of this paper are consistent with the ones in [19]. On the other hand, the results are inconsistent with the theoretical model of [22], as there is no higher risk-premium when the president is from a left-wing party. Also, the fact that the same party is in office for long periods, as in Mexico, is inconsistent with the political cycles observed in the model. Only the negative market reaction after the election of a left-wing presidential candidate is consistent with their model.

Some potential extensions of this research are analyzing the main macro variables under different governments, particularly in the Christian-Secular dimension. Moreover, the presence of Christian parties outside Latin America would justify a study of those cases (e.g., Germany). The extension of the analysis in the religious dimension is particularly important as the regimes under Christian parties are less common than under Secular parties, and they remain more the exception than the norm in the sample. Also, the Christian parties included in the article, following [28], are related to Christian Democrats but not to Evangelical Christianity parties, such as Partido Encuentro Social in Mexico. Thus, we cannot extrapolate the results to these parties that have not achieved the presidency yet.

Another possible extension is analyzing potentially unforeseen relationships with nonpolitically motivated variables. For example, as regulated industries are more likely to be targets of different political platforms, it is expected that these industries would be more affected by the changes of the president in office.

Finally, an additional consideration concerns the ideological heterogeneity of governing coalitions. While the empirical analysis accounts for partisan control of Congress, coalitions such as the *Concertacion* combined parties spanning the center-left spectrum, including Christian Democratic (centrist) and more explicitly leftist parties. This internal ideological diversity could influence policy outcomes, as presidents may face constraints from coalition partners with differing preferences. For instance, while Lagos came to power through an alliance that included the Christian Democrats, potentially moderating policy choices relative to a more ideologically homogeneous left coalition, was classified as left, but Aylwin and Frei, Christian Democrats, where classified as center governments. We think that future work could explicitly measure coalition heterogeneity (e.g., ideological dispersion of coalition parties), and its evolution over time, to assess whether broader or more centrist coalitions produce systematically different outcomes. We are thankful to a reviewer for this comment and suggestion.

## 8. Conclusion

Taken together, the empirical evidence provides a coherent picture of how partisan politics influences stock returns in Latin American countries with strong presidential systems. First, unlike the well-documented Democratic Party premium in the United States, we find no evidence of a left-wing premium in Latin America. Instead, firm-level stock returns are highest during centrist presidencies, while returns under left- and right-wing governments are statistically lower. These results suggest underreaction to the political election of a centrist president. Second, stock market reaction evidence shows that financial markets respond negatively to the election of left-wing presidential candidates relative to right-wing candidates, indicating that investors perceive these electoral outcomes differently even though this does not translate into a persistent left-wing return premium. Third, introducing the religious dimension of politics reveals a novel and remarkably consistent pattern: governments led by Christian parties are associated with higher average stock returns, positive market reactions following electoral victories, lower stock price crash risk, lower return volatility, lower economic policy uncertainty in most specifications, and higher firm profitability. These findings suggest that the superior stock market performance under Christian parties is unlikely to reflect compensation for higher risk and is instead more consistent with improved expected cash flows and market underreaction to political information.

This paper studies the impact of the president in office on stock returns in a sample of countries under a strong presidential system. Extensive literature shows a very distinct difference in the performance of the stock markets under Republican and Democratic presidents in the U.S. However, the results are not found in the international context. In this paper, we select a unique sample, including countries with democratic systems, strong presidentialism, and noticeable differences in their economic policies. This remarkably contrasts with previous international studies focused on developed countries with parliamentary regimes.

In this study, we show that the president’s party helps explain the time series of stock returns. Market returns are higher when the president is from a Centrist party than left and right-wing governments. There is no consistent, significant difference in market returns between left-wing and right-wing presidencies; thus, the results are inconclusive in this respect. The market reaction the day after the election is consistent with previous results and significantly lower for elections when the president elected is to the left of the candidate ending up in second place. Several risk measures are used with inconsistent results to explain the differences in market returns. Also, the control of the congress seems relevant, particularly for right-wing governments, that may seem to have a harder time implementing their policies.

Second, the religious dimension of the party in office is explored. Governments with a Christian party in power have consistently superior performance than when secular parties are in charge. The stock returns are higher, the ROE is higher, and the risk is lower during the presidential terms under a leader from a Christian party. These results are consistent with the cash flow-based explanation.

Collectively, these results suggest that the party of the president in office is economically relevant for the performance of the stock markets, not only in the more studied right-left wing dimension but also in the religious dimension. Moreover, the higher returns for the terms when the president is from a Christian party are not explained by any measure of risk. These results support an underreaction to the political variable.

## Supporting information

S1 File Appendix. (DOCX)
