# Supplementary material for: Partisan politics and stock returns under strong presidential regimes: Religion and politics
Source: PLoS One. 2026 Oct 30;21(10):e0359627. doi: 10.1371/journal.pone.0359627 (PMC13722321; doi:10.1371/journal.pone.0359627)
Supplement: Supplementary file 1 [file pone.0359627.s001.docx]

## Appendix

**Table A1**

This table shows the classification of Latin American parties that is used in this study and updated from [28]. When a party was not included in [28], the additional source and/or reasoning of the classification in included.

| **Country** | **Party** | **Right/Left** | **Christian/Secular** | **Source/Explanation** |
| --- | --- | --- | --- | --- |
| Argentina | Union Cívica Radical | Center | Secular | [28] |
| Argentina | Partido Justicialista | Other | Other | [28] |
| Argentina | Propuesta Republicana (*) | Center-Right | Secular | Not in the final sample |
| Brazil | Partido do Movimento Democrático Brasileiro | Center | Secular | [28] |
| Brazil | Partido de Reconstrução Nacional | Personalist | Personalist | [28] |
| Brazil | Partido da Social Democracia Brasileira | Center-Left | Secular | [28] |
| Brazil | Partido dos Trabalhadores | Left | Secular | [28] |
| Chile | Dictadura Militar (**) | Right | Secular | Pinochet was supported by right-wing parties in the 1988 election. |
| Chile | Partido Demócrata Cristiano | Center | Christian | [28] |
| Chile | Partido por la Democracia | Center-Left | Secular | [28] |
| Chile | Partido Socialista | Center-Left | Secular | [28] |
| Chile | Renovación Nacional | Right | Secular | [28] |
| Colombia | Partido Conservador Colombiano | Center-Right | Secular | [28] |
| Colombia | Partido Liberal Colombiano | Center | Secular | [28] |
| Colombia | Primero Colombia (*) | Center-Right | Secular | Wikipedia, consistent with the current party of its leader, and consistent with the V-Party database. |
| Colombia | Partido Social de Unidad Nacional (*) | Center | Secular | colombia.vota.com, consistent with the V-Party database. |
| Mexico | Partido Revolucionario Institucional | Center-Right | Secular | [28] |
| Mexico | Partido Acción Nacional | Center-Right | Christian | [28] |
| Peru | Partido Aprista Peruano | Center-Left | Secular | [28] |
| Peru | Cambio 90, Perú 2000 | Personalist | Personalist | [28] |
| Peru | Acción Popular (*) | Center | Secular | Wikipedia, consistent with the coalition Center Front formed in 2006, V-Party database. |
| Peru | Perú Posible (*) | Center | Secular | Wikipedia, consistent with the V-Party database. |
| Peru | Partido Nacionalista Peruano (*) | Center-Left | Secular | academia-lab.com, Wikipedia, consistent with the V-Party database. |
| Peru | Peruanos Por el Kambio (*) | Center-Right | Secular | Wikipedia, consistent with the V-Party database. |
| Venezuela | Acción Democrática | Center-Left | Secular | Not in the final sample |
| Venezuela | Coalition (Velásquez): COPEI & Acción Democrática (***) | Center | Christian - Secular | Not in the final sample |
| Venezuela | Convergencia Nacional | Personalist | Personalist - Christian | Not in the final sample |
| Venezuela | Movimiento Quinta República/ Partido Socialista Unificado de Venezuela (*) | Left | Secular | Not in the final sample |

(*) A party that did not exist before 1997

(**) Military dictatorship between September 1973 and March 1990. This government was supported mainly by the right-wing. Parties were suppressed until 1988. After 1988, the right-wing parties officially supported a democratic election of the dictatorship president Augusto Pinochet.

(***) President Velásquez was independent but supported by the COPEI (Center-Righ – Christian) and Acción Democrática (Center-Left – Secular)

**Table A2	Pooled-OLS regressions and the party of the President in office. Left to Right dimension.**

This table reports the results of the following pooled-OLS regressions. $R_{i,t}=\gamma_{1}{LL}_{t}+\gamma_{2}{CC}_{t}{+\gamma}_{3}{RR}_{t}{+\gamma}_{4}{CV}_{t}+\epsilon_{i,t}$. $R_{i}$ refers to the firm $i$ monthly excess returns in US dollars, and $LL$, $CC$, and $RR$ are the dummy variables for Left-wing, Center, and Right-wing parties respectively. $CV$ is the set of control variables for Brazil, Chile, Colombia, and Mexico including Industrial Production (IP), Inflation, the Economic Policy Index (EPU) as in section 5.1, and the daily volatility as calculated in section 5.3.2. The sample covers the period from July 1994 to December 2017 for Brazil, January 1997 to December 2017 for Chile, January 2000 to December 2016 for Colombia, and April 1996 to December 2017 for Mexico, according to data availability. The first row represents the coefficients; the second row reports the t-statistics. ***, **, and * indicate significance at the 1%, 5%, and 10% levels, respectively.

|  | Ri-Rf |
| --- | --- |
| VARIABLES | US dollars |
|  |  |
| LL | 0.0313*** |
|  | (30.05) |
| RR | 0.0249*** |
|  | (22.77) |
| CC | 0.0282*** |
|  | (17.81) |
| **LL-RR** | **0.0064***** |
| **LL-CC** | **0.0031**** |
| **RR-CC** | **-0.0033**** |
|  |  |
| IP | -0.0004* |
|  | (-1.88) |
| Inflation | 0.0094*** |
|  | (9.66) |
| EPU | -0.000038*** |
|  | (-5.44) |
| Volatility | -1.0460*** |
|  | (-24.21) |
|  |  |

**Table A3 Pooled-OLS regressions and the party of the President in office. Secular and Christian dimension**

This table reports the results of the following pooled-OLS regressions. $R_{i,t}=\delta_{1}{SS}_{t}+\delta_{2}{XX}_{t}{+\delta}_{3}{CV}_{t}+\epsilon_{i,t}$. $R_{i}$ refers to the firm $i$ monthly excess returns in US dollars, and $SS$ and $XX$ are the dummy variables for Secular and Christian parties respectively. $CV$ is the set of control variables for Brazil, Chile, Colombia, and Mexico including Industrial Production (IP), Inflation, the Economic Policy Index (EPU) as in section 5.1, and the daily volatility as calculated in section 5.3.2. The sample covers the period from July 1994 to December 2017 for Brazil, January 1997 to December 2017 for Chile, January 2000 to December 2016 for Colombia, and April 1996 to December 2017 for Mexico, according to data availability. The first row represents the coefficients; the second row reports the t-statistics. ***, **, and * indicate significance at the 1%, 5%, and 10% levels, respectively.

|  | Ri-Rf |
| --- | --- |
| VARIABLES | US dollars |
|  |  |
| SS | 0.0284*** |
|  | (29.67) |
| XX | 0.0279*** |
|  | (20.42) |
| **SS-XX** | **0.0005** |
|  |  |
| Control Variables: |  |
| IP | -0.0004* |
|  | (-1.90) |
| Inflation | 0.0095*** |
|  | (9.77) |
| EPU | -0.000038*** |
|  | (-5.57) |
| Volatility | -0.9918*** |
|  | (-23.33) |
|  |  |

**Appendix A4 Central Bank Independence**

Below is the timeline regarding when the Central Banks of the countries covered in the sample achieve their independency. Central Bank Independence is treated as a dummy based on the initial legal or constitutional reform.

***Chile:*** *Central Bank becomes independent in October 1989 based on Law 18840. The new independent board started in December of the same year.*

*For additional information:* [*https://www.bcentral.cl/en/the-bank/corporative-goverment/history*](https://www.bcentral.cl/en/the-bank/corporative-goverment/history)*,* [*https://www.bcentral.cl/en/web/banco-central/content/-/detalle/ley-organica-constitucional-del-banco-central-de-chile-ley-n.c2b0-18.840-3*](https://www.bcentral.cl/en/web/banco-central/content/-/detalle/ley-organica-constitucional-del-banco-central-de-chile-ley-n.c2b0-18.840-3)

***Colombia:*** *The Central Bank of Colombia (Banco de la República) became legally and constitutionally independent in 1991, with the adoption of the Colombian Constitution of 1991 (Article 371).*

*For additional information:* [*https://www.banrep.gov.co/en/node/7622*](https://www.banrep.gov.co/en/node/7622)

***Peru:*** *The Central Bank of Peru (Banco Central de Reserva del Perú) became legally and constitutionally independent in 1993, with the enactment of the 1993 Constitution (Article 84).*

*For additional information:* [*https://www.constituteproject.org/constitution/Peru_2021*](https://www.constituteproject.org/constitution/Peru_2021) *and* [*https://www.bcrp.gob.pe/transparencia/datos-generales/marco-legal/capitulo-v-de-la-constitucion.html*](https://www.bcrp.gob.pe/transparencia/datos-generales/marco-legal/capitulo-v-de-la-constitucion.html)

***Mexico:*** *The Central Bank of Mexico (Banxico) has been independent since April 1, 1994, when a key reform to Article 28 of the Mexican Constitution came into effect, granting it constitutional autonomy.*

*For additional information:* [*https://www.banxico.org.mx/getting-to-know-banco-de-mexico/history-hierarchical-history-.html*](https://www.banxico.org.mx/getting-to-know-banco-de-mexico/history-hierarchical-history-.html)

***Brazil:*** *The Central Bank of Brazil is independent from the Federal Government since 25 February 2021 (Federal Complementary Law No. 179)*

*For additional information:* [*https://www.bcb.gov.br/detalhenoticia/517/noticia*](https://www.bcb.gov.br/detalhenoticia/517/noticia)
